# The Influence of Hypertensive Therapies on Circulating Factors: Clinical Implications for SCFAs, FGF21, TNFSF14 and TNF-α

**DOI:** 10.3390/jcm9092764

**Published:** 2020-08-26

**Authors:** Aaron L. Magno, Lakshini Y. Herat, Márcio G. Kiuchi, Markus P. Schlaich, Natalie C. Ward, Vance B. Matthews

**Affiliations:** 1 Research Centre, Royal Perth Hospital, Perth, WA 6000, Australia; aaron.magno@uwa.edu.au; 2 Dobney Hypertension Centre, School of Biomedical Science—Royal Perth Hospital Unit, University of Western Australia, Crawley, WA 6009, Australia; lakshini.weerasekera@uwa.edu.au; 3 Dobney Hypertension Centre, School of Medicine—Royal Perth Hospital Unit, University of Western Australia, Crawley, WA 6009, Australia; marcio.galindokiuchi@uwa.edu.au (M.G.K.); markus.schlaich@uwa.edu.au (M.P.S.); 4 Department of Cardiology and Department of Nephrology, Royal Perth Hospital, Perth, WA 6000, Australia; 5 School of Public Health, Curtin University, Perth, WA 6102, Australia; natalie.ward@uwa.edu.au; 6 School of Medicine, University of Western Australia, Perth, WA 6009, Australia

**Keywords:** human, hypertension, short chain fatty acids, FGF21, TNFSF14, TNF-α

## Abstract

Studying the role of circulatory factors in the pathogenesis of diseases has been key to the development of effective therapies. We sought to examine the effect of antihypertensive therapies on numerous circulatory factors including short chain fatty acids and growth factors in a human cohort. A subset of participants from an earlier study was characterized by their hypertensive and/or treatment status and separated into three groups: (i) normotensives; (ii) untreated hypertensive and (iii) treated hypertensive subjects. Circulating levels of short chain fatty acids, FGF21 and TNF superfamily members were measured as part of this study. Both F2-isoprostane and circulating lipid levels were reanalysed as part of this current study. We found that antihypertensive treatment increased butyrate levels and decreased acetate levels to levels similar to normotensives. We also found that antihypertensive treatments reduced levels of circulating FGF21, TNFSF14 and TNF-α. In conclusion, we identified several circulatory factors that are altered in hypertension.

## 1. Introduction

In 2013, approximately 6 million premature cardiovascular disease (CVD) deaths in individuals aged between 30 and 70 years of age occurred globally based on the Global Burden of Disease Study data set [1]. If trends continue, the number of premature CVD deaths is estimated to rise to close to 8 million. As such, it is imperative that means to reduce the risk of CVD be developed [1]. Hypertension has been shown to be closely associated with an increased risk of CVD independent of other CVD risk factors [2]. With over 30% of the global population being affected by elevated blood pressure (BP) [3], it is a prime target for intervention to reduce the number of CVD deaths worldwide. However, the regulation of BP is complex and influenced by several interrelated factors and mechanisms. These include the renin–angiotensin–aldosterone system (RAAS), renal pathways involved in the regulation of sodium and volume balance, mechanisms regulating peripheral resistance, endothelial dysfunction and the sympathetic nervous system (SNS) [4].

The recent clinical trials, Empagliflozin Cardiovascular Outcome Event Trial in Type 2 Diabetes Mellitus Patients (EMPA-REG OUTCOME) [5] and Canagliflozin Cardiovascular Assessment Study (CANVAS) [6], which investigated the use of sodium glucose cotransporter 2 (SGLT2) inhibitors in patients with type 2 diabetes, have shown that these inhibitors have additional cardiovascular protective properties. In our recent preclinical studies, we explored the interaction between sympathetic hyperactivity and SGLT2 regulation using a neurogenic model of hypertension, the BPH/2J mouse (Schlager mouse) [7,8]. We found that the downregulation of the SNS via SGLT2 inhibition reduced hypertension in this model. These data indeed suggest that the SGLT2 inhibitor, Dapagliflozin, may improve hypertension via sympathoinhibition [7]. To further understand the mechanisms by which the SGLT2 inhibitor acted, we examined changes in a number of short chain fatty acids (SCFAs) and numerous circulatory factors in our preclinical model [7]. To determine if the effects of SGLT2 inhibition on hypertension and circulatory factors observed in our mouse model were similar to those of established antihypertensive treatments, we re-examined a human cohort consisting of normotensive, untreated hypertensive and treated hypertensive subjects, in which oxidative stress had previously been studied [9]. We chose to analyse the levels of the SCFAs butyrate, acetate and propionate in our human cohort, as the supplementation of each has previously been shown to prevent the development of hypertension in rodent models [10]. Clinical studies have shown an association between hypertension and the circulating levels of fibroblast growth factor 21 (FGF21) [11,12,13], tumour necrosis factor superfamily member 14 (TNFSF14) [14] and tumour necrosis factor-α (TNF-α) [15,16]. Here, we sought to examine the effect of antihypertensive drugs on these circulatory factors.

## 2. Experimental Section

### 2.1. Subjects

A total of 155 hypertensive subjects and 40 normotensive control subjects were recruited from the Perth general population to the Royal Perth Hospital Unit of the School of Medicine and Pharmacology at the University of Western Australia [9]. Exclusion criteria included: previous coronary or cerebrovascular event <6 months, heart failure, premenopausal women, the use of oral contraception, the use of nitrate medication, and body mass index (BMI) >35 kg/m^2^. For this analysis, a history of cardiovascular disease or additional cardiovascular disease risk factors were used as additional exclusion criteria. Specimens were drawn from a larger previously collected cohort of controls and hypertensives, both on and off antihypertensive treatment and with and without additional CVD risk factors. Those with existing risk factors were eliminated for the sake of clarity. Available remaining samples underwent the analysis of circulating SCFAs (55 hypertensive subjects and 10 normotensive control subjects) and the analysis of circulatory factors (30 hypertensive subjects and 18 normotensive control subjects). As part of the SCFA analysis, the serum levels of butyrate, acetate and propionate were measured. The circulatory factors measured as part of the biochemical analysis were FGF21, TNFSF14, tumour necrosis factor-α (TNF-α), insulin and creatinine. Levels of glucose, cholesterol, triglycerides and F2-isoprostanes measured as part of the previous study [9] underwent reanalysis with the additional exclusion criteria. All volunteers ceased any vitamin, antioxidant, or fish oil supplements for a minimum of 4 weeks prior to entry into the study. Where possible, all prescribed medications were taken as normal on the morning of each visit. Subjects initially had their BMI measured and were fitted with a 24 h ambulatory BP monitor (ABPM, Spacelabs 90207, NSW, Australia). The next day, the BP monitor was removed and a fasting blood sample was collected. The World Health Organization—International Society of Hypertension Guidelines were used to define normotensive subjects as having a mean 24 h BP <125/80 mmHg. Untreated hypertensive subjects were included if their mean 24 h systolic BP (SBP) was ≥135 mmHg and had never been treated. Treated hypertensive subjects were included on the basis of a prior physician diagnosis of essential hypertension and currently taking one or more antihypertensive medications for ≥3 months. Treatments included up to three of the following medications; angiotensin-converting enzyme inhibitors, angiotensin II receptor blockers, β-blockers, calcium channel entry blockers and diuretics. The study was approved by the Royal Perth Hospital Human Ethics Committee. Written informed consent was obtained before inclusion in the study.

### 2.2. Short-Chain Fatty Acids Analysis

The concentration of the SCFAs butyrate, acetate and propionate in the serum samples isolated from the cohort were assessed using gas chromatography-mass spectrometry as previously described [17,18]. Data were calculated as nanomoles per litre serum.

### 2.3. Enzyme-Linked Immunosorbent Assays

Serum was analysed for FGF21, TNFSF14, TNF-α and insulin, using the appropriate enzyme-linked immunosorbent assay (ELISA) kits according to the manufacturer’s respective instructions (Duoset human FGF21 ELISA kit (Cat# DY2539, R&D systems, Inc., Minneapolis, MN, USA), Duoset human TNFSF14 ELISA kit (Cat# DY664, R&D systems) and Duoset human TNF-α ELISA kit (Cat# DY210, R&D systems)).

### 2.4. Oxidative Stress Biomarkers

Plasma and urinary F2-isoprostanes were analysed as previously described using a Hewlett-Packard HP 5890 Series II Plus gas chromatograph (Hewlett-Packard, Roseville, CA, USA), coupled to an HP 5989B Mass Spectrometer (Hewlett-Packard) operated in the selective ion monitoring mode and using HP Chem-Station G1034C Revision C.03.03 Software [19].

### 2.5. Biochemistry

Fasting serum cholesterol, triglyceride, high-density lipoproteins (HDL), low-density lipoproteins (LDL), insulin and glucose were analysed using standard enzymatic methods (Boehringer Mannheim, San Francisco, CA, USA) with a fully automated analyser (Hitachi 917) in the Department of Clinical Biochemistry at Royal Perth Hospital [9].

### 2.6. Statistical Analysis

Data were analysed using one-way ANOVA with Dunnett’s post hoc comparison to compare the means of the three groups. Quantitative data are presented as the mean ± SE. Data were deemed significant when *p* < 0.05. Graphs were produced with GraphPad Prism 8 (GraphPad Software Inc., San Diego, CA, USA).

## 3. Results

### 3.1. Characterization of Study Population

Subpopulations of a previously examined cohort [9] were characterized by their hypertensive and/or treatment status and separated into three groups: normotensive, untreated hypertensive and treated hypertensive subjects. Treated hypertensive subjects were on up to three different antihypertensive agents. These included angiotensin-converting enzyme inhibitors, angiotensin II receptor blockers, β-blockers, calcium channel entry blockers and diuretics. Samples from these participants were subjected to SCFA or biochemical analysis. Of the subjects receiving antihypertensive treatments that had their samples measured for SCFA levels, 15 were using angiotensin-converting enzyme inhibitors, 13 angiotensin II receptor blockers, six β-blockers, eight calcium channel entry blockers and 11 diuretics. For biochemical analysis, eight were using angiotensin-converting enzyme inhibitors, five angiotensin II receptor blockers, three β-blockers, four calcium channel entry blockers and two diuretics. The key characteristics of the participants are presented in Table 1 (SCFA analysis) and Table 2 (biochemical analysis). In the cohort that underwent the SCFA analysis, the systolic BP (SBP) and diastolic BP (DBP) of untreated hypertensive subjects was higher than those of the normotensive subjects. The SBP and DBP of the untreated hypertensive population was significantly higher than both the normotensive and treated hypertensive populations involved in the biochemical analysis. This indicates that the antihypertensive agents used, lowered blood pressure of the hypertensive patients effectively. It should be noted that the treated hypertensive subjects were significantly older than the normotensive and untreated hypertensive subjects.

### 3.2. Effects of Antihypertensive Treatment on the Production of SCFAs

**Figure 1:**
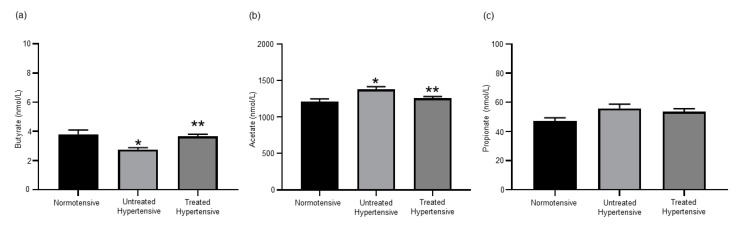
Short chain fatty acid serum levels were altered in response to antihypertension treatments. (**a**) Serum butyrate concentrations. * Normotensive vs untreated hypertensive *p* = 0.003, ** untreated hypertensive vs. treated hypertensive *p* = 0.001; (**b**) serum acetate concentrations. * Normotensive vs. untreated hypertensive *p* = 0.007, ** untreated hypertensive vs. treated hypertensive *p* = 0.013; and (**c**) serum propionate concentrations. *n* = 10–39 subjects/group. All data are presented as the mean ± SEM.

As we had found that the administration of Dapagliflozin to hypertensive mice lowered their BP and altered their SCFA levels [7], we examined whether antihypertensive treatments would also affect the SCFA levels in a human cohort. The serum of the cohort was analysed for the levels of the SCFAs butyrate, acetate and propionate (Figure 1). The levels of butyrate detected in the normotensive (3.80 ± 0.30 nmol/L) and treated hypertensive (3.67 ± 0.13 nmol/L) populations were similar and significantly higher than those in the untreated hypertensive (2.76 ± 0.12 nmol/L) population (*p =* 0.003). Acetate levels detected in the normotensive (1209.00 ± 38.63 nmol/L) and treated hypertensive (1256.38 ± 22.58 nmol/L) populations were also similar. The level of acetate in the untreated hypertensive (1377.81 ± 36.58 nmol/L) population was significantly higher than that seen in the other populations (*p =* 0.007). The levels of propionate had a trend to be higher in the untreated hypertensive (55.84 ± 2.92 nmol/L) group in comparison to the normotensive (47.27 ± 2.09 nmol/L) group. The concentration of serum propionate in the treated hypertensive group was 53.59 ± 2.01 nmol/L.

### 3.3. Antihypertensive Treatment Reduces Expression of FGF21

**Figure 2:**
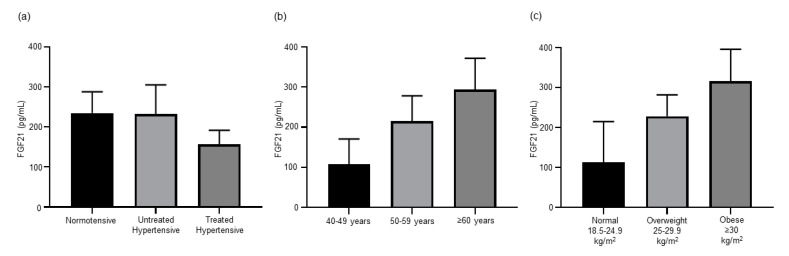
(**a**) Antihypertension treatments decreased the level of FGF21 in hypertensive patients (*n* = 12–18). (**b**) Circulating levels of FGF21 increase with age (*n* = 5–14) and (**c**) BMI (*n* = 3–21). All data are presented as the mean ± SEM. FGF21, fibroblast growth factor 21.

In our previously published experiments with hypertensive mice, Dapagliflozin significantly reduced BP and FGF21 levels [7]. Circulating levels of FGF21 were lower, but not significantly different, in the treated hypertensive (156.9 ± 34.61 pg/mL) population than both the normotensive (234.83 ± 52.85 pg/mL) and untreated hypertensive (232.7 ± 72.44 pg/mL) populations (Figure 2a). As antihypertensive treatments decrease the level of circulating FGF21, this treatment group was excluded from analysis to better understand the role of FGF21 in hypertension irrespective of antihypertensive treatment. The concentration of FGF21 in serum was similar irrespective of differences in BP (Figure S1). Subjects with optimal to normal SBP (≤129 mmHg) (264.05 ± 72.05 pg/mL) had similar FGF21 levels to those with high-normal to high SBP (≥130 mmHg) (239.97 ± 66.90 pg/mL). The FGF21 levels in the optimal to normal DBP (≤84 mmHg) population (243.11 ± 59.01 pg/mL) were comparable to those in the high-normal to high DBP (≥85 mmHg) population (264.18 ± 88.14 pg/mL). As the treated hypertensive group was significantly older than the normotensive and untreated hypertensive groups, age was examined as a possible factor contributing to the non-significant trend of a lower serum FGF21 concentration in the treated hypertensive group. FGF21 levels steadily increased with age, 40–49 years (107.00 ± 63.35 pg/mL), 50–59 years (215.42 ± 62.64 pg/mL) and ≥60 (315.30 ± 73.53 pg/mL) (Figure 2b), but there was no significant difference between the three age groups. A non-significant trend increase in FGF21 levels was also observed with increased BMI, as subjects with a normal (18.6–24.9 kg/m^2^) BMI (112.68 ± 102.22 pg/mL) had lower FGF21 levels than those that were overweight (25–29.9 kg/m^2^) (228.03 ± 53.62 pg/mL) or obese (≥30 kg/m^2^) (315.42 ± 80.05 pg/mL) (Figure 2c).

### 3.4. Antihypertensive Treatment Reduces Levels of TNF Superfamily Members

**Figure 3:**
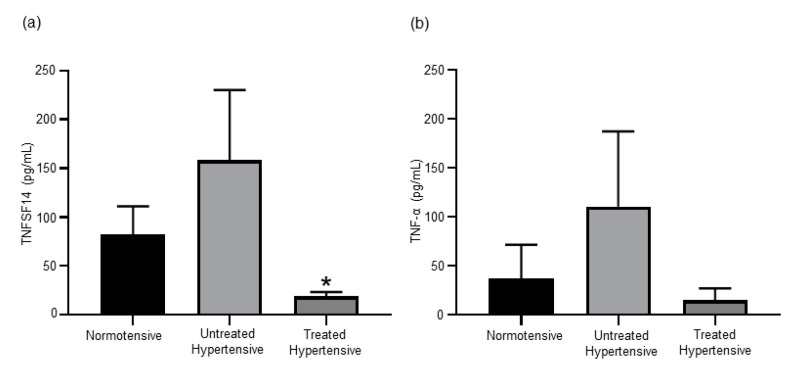
(**a**) The circulating levels of TNFSF14 was higher in the untreated hypertensive subjects. * Untreated hypertensive vs. treated hypertensive *p* = 0.033. (**b**) Serum TNF-α concentrations are elevated in untreated hypertensive subjects. *n*= 10–39 subjects/group. All data are presented as the mean ± SEM. TNFSF14, tumour necrosis factor superfamily member 14.

We examined the comparative levels of TNF family members in this human cohort as increases in TNFSF14 [20] and TNF-α [21] levels have both been shown to be associated with elevated BP (Figure 3). The circulating levels of TNFSF14 were higher in the untreated hypertensive (158.59 ± 71.77 pg/mL) group in comparison to the normotensive (82.29 ± 28.76 pg/mL) group. The treated hypertensive population (18.78 ± 4.61 pg/mL) had a significantly lower level of TNFSF14 than the untreated hypertensive (*p* = 0.033) population. A similar trend was detected when circulating TNF-α was measured, with the treated hypertensive group being lower than both the normotensive and untreated hypertensive groups.

### 3.5. Effects of Antihypertensive Treatment on Oxidative Stress Biomarkers

There is evidence that oxidative stress plays a role in the pathogenesis of hypertension [22]. The biological marker of oxidative stress, F2-isoprostane levels, has been observed to be higher in hypertensive patients [23,24]. The analysis of this subset of the original cohort is presented in Figure S2. There were no significant differences between the plasma F2-isoprostane levels of the normotensive (3356.00 ± 428.05 pmol/L), untreated hypertensive (3185.67 ± 351.64 pmol/L) and treated hypertensive (2776.72 ± 285.66 pmol/L) groups. This was also observed when urinary F2-isoprostane levels were measured in the normotensive (1816.00 ± 911.46 pmol/24 h), untreated hypertensive (1485.00 ± 453.80 pmol/24 h) and treated hypertensive (1020.05 ± 258.05 pmol/24 h) populations.

### 3.6. Insulin Resistance in Hypertensive Subjects

**Figure 4:**
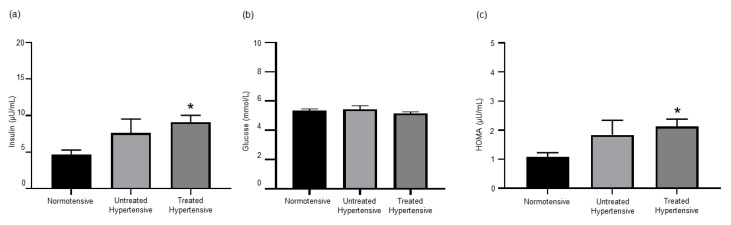
(**a**) Insulin levels are elevated in hypertensive patients. * normotensive vs. treated hypertensive *p* = 0.027; (**b**) serum glucose concentrations; and (**c**) insulin resistance index. * normotensive vs. treated hypertensive *p* = 0.05. *n* = 11–18 subjects/group. All data are presented as the mean ± SEM. HOMA, homeostatic model assessment.

Both insulin and glucose levels were measured to determine the level of insulin resistance in this cohort (Figure 4). Insulin levels were higher in both hypertensive groups, untreated hypertensive (7.63 ± 1.89 µU/mL) and treated hypertensive (9.11 ± 0.93 µU/mL), compared to the normotensive (4.70 ± 0.57 µU/mL) group. There was no significant variation in the glucose levels of the three groups, normotensive (5.42 ± 0.26 mmol/L), untreated hypertensive (5.14 ± 0.12 mmol/L) and treated hypertensive (5.34 ± 0.12 mmol/L). As such, the insulin resistance score as determined by the homeostatic model assessment (HOMA) of the treated hypertensive (2.13 ± 0.25 µU/mL) population was significantly higher than that of the normotensive (1.07 ± 0.15 µU/mL) population (*p =* 0.05). The insulin resistance score was also greater in the untreated hypertensive (1.84 ± 0.50 µU/mL) group than in the normotensive group.

### 3.7. Kidney Dysfunction in Hypertensive Subjects

**Figure 5:**
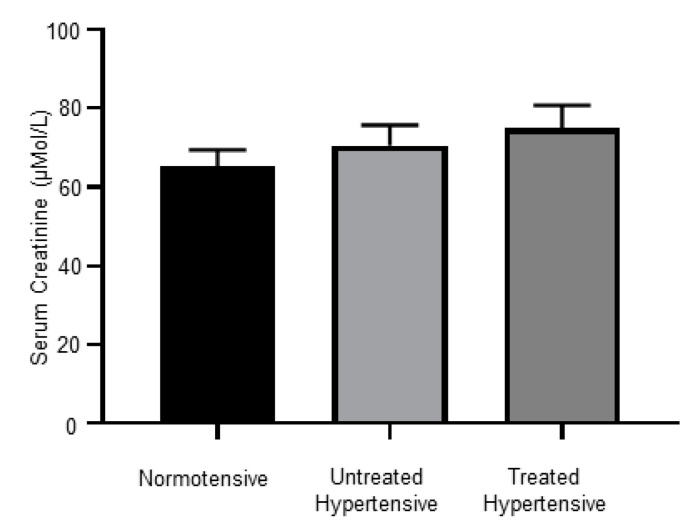
Serum creatinine levels; *n* = 12–16 subjects/group. All data are presented as the mean ± SEM.

There were no significant differences between the serum creatinine levels of the normotensive (65.27 ± 4.18 µMol/L), untreated hypertensive (70.58 ± 5.20 µMol/L) and treated hypertensive (74.94 ± 5.87 µMol/L) groups, Figure 5.

### 3.8. Antihypertensive Treatments Impact on Circulating Lipid Levels

Cholesterol, triglyceride and lipoprotein levels were measured in the fasting serum taken from the cohort (Figure S3). Total cholesterol levels were lower in the hypertensive population, untreated hypertensive (5.10 ± 0.15 mmol/L) and treated hypertensive (4.91 ± 0.23 mmol/L) than in the normotensive (5.56 ± 0.24 mmol/L) population. This trend was also seen in the cohort’s LDL levels, normotensive (3.68 ± 0.20 mmol/L), untreated hypertensive (3.14 ± 0.19 mmol/L) and treated hypertensive (3.15 ± 0.21 mmol/L). However, HDL levels in the treated hypertensive (1.20 ± 0.07 mmol/L) population were significantly lower than those of the normotensive (1.47 ± 0.07 mmol/L) population (*p* = 0.025). The HDL levels of the untreated hypertensive (1.42 ± 0.09 mmol/L) group were similar to that of the normotensive group. Triglyceride levels were higher in the hypertensive population, untreated hypertensive (1.17 ± 0.24 mmol/L) and treated hypertensive (1.18 ± 0.17 mmol/L) than in the normotensive (0.93 ± 0.12 mmol/L) population.

## 4. Discussion

Studying the impact of relevant circulatory factors in the pathogenesis of numerous diseases has been instrumental in the development of effective therapies. For example, monitoring a central sympathetic outflow and circulating norepinephrine levels in hypertensive patients highlighted the role of the SNS as an important therapeutic target. This led to the development of moxonidine, a drug that lowers BP by suppressing SNS activity [25]. Our current study highlights for the first time that TNFSF14 levels are reduced with antihypertensive therapy. We also examined the impact of antihypertensive treatments on a variety of other circulatory factors including SCFAs, growth factors and cytokines. We showed that the elevation of the SCFA butyrate, is a reliable marker of cardiovascular health after antihypertensive therapy. We also found that antihypertensive treatments decrease the growth factor FGF21 and the cytokine TNF-α, perhaps highlighting the beneficial impact of antihypertensive therapy on subclinical inflammation.

There is great interest in the role that the gut microbiome plays in hypertension and the impact of antihypertensive treatments on the gut microbiome [26]. Short chain fatty acids are a product of the gut microbiota. These products include butyrate, acetate and propionate and contribute to immune and inflammatory responses, as well as the control of lipid and glucose homeostasis. Studies from our group and others indicate that numerous drug classes alter the gut microbiome [17,18]. In our preclinical mouse model of hypertension, we found that the use of an SGLT2 inhibitor to lower BP also elevated the levels of butyrate and acetate found in cecum contents [7]. Serum butyrate levels were also elevated in diabetic mice treated with SGLT2 inhibitors [27]. Preclinical studies have shown that treatment with butyrate, acetate and propionate can lower BP [28,29]. In our human cohort, antihypertensive treatments increased the level of butyrate in hypertensive patients to levels similar to that of normotensive subjects. Conversely, acetate levels in hypertensive patients were lowered by antihypertensive treatments to levels similar to those detected in normotensive subjects. This is the opposite trend we observed in our hypertensive mouse model [7]. However, in mice on a high fat diet, it was found that the supplementation with isoquercetin and inulin lowered serum acetate levels to that of mice on normal chow [18].

While FGF21 is produced in many tissues including muscle, liver, pancreas and heart, a major source of FGF21 is adipose tissue [30]. It has been shown to effect a wide range of metabolic functions including glucose and lipid metabolism and insulin sensitivity [31]. Evidence from clinical studies identified an association between elevated circulating FGF21 and hypertension [11,12,13]. Experiments in several mouse models of hypertension demonstrated that FGF21 can prevent or ameliorate hypertension [32,33,34]. This suggests a protective role for FGF21 against hypertension. In the cohort analysed here, we found serum FGF21 levels to be similar between normotensive and untreated hypertensive subjects. However, antihypertensive treatments lowered the level of circulating FGF21 in hypertensive patients. This outcome reflects our findings using a hypertensive mouse model that documented a reduction in serum FGF21 that coincided with the treatment that successfully reduced BP [7]. In obese women that participated in a 3 month exercise program, a reduction in both BP and FGF21 was observed [35]. The reduction in BP may result in a lesser need for the protective benefits of FGF21 and therefore the level of circulating FGF21 may be concomitantly lowered.

Our data show that serum FGF21 levels increase with age (Figure 2b). This is consistent with other studies that also show an age-dependent increase in FGF21 levels [36,37]. In our study, the treated hypertensive group was significantly older than the other groups (Table 2). The fact that we were able to observe lower FGF21 levels after antihypertensive treatment highlights the powerful effect of treatment on FGF21 regulation that overcomes the effect of age. Our findings support those of others that have identified a positive correlation between FGF21 and BMI (Figure 2c) [38,39,40]. In our study, the patients treated with antihypertensive therapy had a similar BMI (Table 2). Hence, the lower circulating FGF21 in the treated hypertensive group were not a result of weight differences.

Members of the TNF cytokine superfamily have been implicated in the progression and severity of hypertension. In women with preeclampsia, a serious hypertensive condition of pregnancy, an elevated level of circulating TNFSF14 has been detected [14]. It was subsequently shown in mouse models that the administration of TNFSF14 could induce hypertension [20]. In our cohort, we found elevated levels of serum TNFSF14 in untreated hypertensive patients compared to the normotensive subjects, which may be directly contributing to the rise in BP. However, treated hypertensive patients had a significantly lower level of circulating TNFSF14. Interestingly, our group has previously demonstrated in mice that TNFSF14 deficiency results in a decrease in FGF21 expression [41]. This raises the possibility that the lower serum FGF21 levels detected in the treated hypertensive patients may be partially due to the lower TNFSF14 levels. Additionally, serum TNF-α levels have previously been found to be elevated in hypertensive patients [15,16], as was also the case in our cohort. Yoshida et al. demonstrated that hypertension may be reduced after the administration of a TNF-α inhibitor [21]. This study showed that TNF-α may drive increased BP via the activation of the SNS and/or RAAS [21]. We have previously shown in mice on a high fat diet that the administration of an SGLT2 inhibitor can reduce TNF-α levels [42]. Antihypertensive treatment reduced the levels of circulating TNF-α in our cohort, which may represent one mechanism by which these therapies reduce BP.

No significant difference in either plasma or urinary F2-isoprostane levels were identified in the analysis of the original cohort [9]. The further exclusion of patients with a history of cardiovascular disease or other cardiovascular disease risk factors did not reveal any significant difference in the plasma and urinary F2-isoprostane levels. However, the trend of lower F2-isoprostane in the treated hypertensive patients was the same in both the original cohort and the subset presented here. Future studies will aim to increase the cohort sizes as this may assist with obtaining significant differences between groups.

Different antihypertensive treatments have varying effects on glucose metabolism. For example, angiotensin-converting enzyme inhibitors and angiotensin II receptor blockers may exert beneficial effects on glycaemic control. Conversely, β-blockers and diuretics have been associated with the development of glucose intolerance and diabetes [43]. In our study, we found higher insulin resistance in patients receiving antihypertensive treatments but it should be noted that multiple agents have been used, including in combination.

It has been shown that controlling BP with inhibitors of the RAAS is associated with renoprotection [44]. However, when we measured serum creatinine levels, we found no decrease in creatinine levels in the treated hypertensive patients, even though most were on RAAS inhibitors. Renoprotection might be observed in this cohort after a longer course of antihypertensive treatment.

The analysis of circulating lipid levels in this subset of subjects showed that only HDL levels were significantly different between the groups. HDL has been considered to have many benefits for the cardiovascular system [45]. The lower HDL levels we detected in the treated hypertensive patients suggest that higher HDL is not necessarily a marker of good cardiovascular health. Currently there is some controversy regarding the role of HDL in cardiac disease with some studies showing beneficial effects, while others show a promotion of cardiac disease [46]. There is growing evidence that the HDL particle number may be a better marker of cardiovascular disease than HDL cholesterol measurements [47].

We have previously shown that the major neurotransmitter of the SNS, norepinephrine, may increase SGLT2 [42] and ADAM28 [48] expression, which are both associated with metabolic dysfunction. Fu et al. also showed that norepinephrine increases TNF-α [49]. We also demonstrated that ADAM28 promoted TNF-α shedding [50]. It would be interesting to investigate the possibility that norepinephrine also upregulates TNFSF14 levels using THP1 monocytes [51]. We hypothesize that it is increased levels of norepinephrine that is causing both TNF-α and TNFSF14 levels to rise in untreated hypertensive patients where we know SNS is likely hyperactive.

There are several limitations to this preliminary investigation, including a small number of subjects. Multiple factors, such as age, gender, race, BMI, underlying aetiologies and therapies may confound the results in a study with a relatively small cohort, but our results can pave the way for further investigation in larger well defined cohorts to overcome the multiple factors. In particular, age can be a confounding factor. Several of the circulatory factors measured as part of the biochemical analysis have been shown to be age-related. Serum FGF21 levels were found to increase with age [36,37]. The treated hypertensive patients in our study are significantly older but have the lowest trending FGF21 levels (Figure 2a) highlighting the suppressive role of the treatments. Both TNFSF14 [52] and TNF-α [53] have also been shown to increase with age. The lowest level of TNFSF14 and a trending lower level of TNF-α were found in the oldest group in our study, the treated hypertensive subjects (Figure 3). Once again, the treatments suppressed the levels of TNFSF14 and TNF-α. Increasing age decreases insulin levels [54]. Interestingly, the highest level of insulin was detected in the treated hypertensive group.

We assessed a limited number of circulatory factors in our current study. It would be of interest to identify a broader range of clinically relevant metabolic cytokines and growth factors in independent cohorts in future studies.

## Figures and Tables

**Table 1:** Characteristics of the participants involved in short chain fatty acid (SCFA) analysis.

Parameter	Normotensive	Untreated Hypertensive	Treated Hypertensive
Gender (M/F)	3/7	11/4	22/17
Age (years)	54.50 ± 2.15	55.60 ± 2.97	58.87 ± 1.40
BMI (kg/m^2^)	27.38 ± 1.11	26.70 ± 0.72	26.64 ± 0.58
SBP (mmHg)	130.40 ± 2.40	138.13 ± 2.30	131.36 ± 1.90
DBP (mmHg)	75.40 ± 6.89	82.47 ± 2.37	80.21 ± 1.47

Values are the means ± SEM. BMI, body mass index; SBP, systolic blood pressure; DBP, diastolic blood pressure.

**Table 2:** Characteristics of the participants involved in the biochemical analysis.

Parameter	Normotensive	Untreated Hypertensive	Treated Hypertensive
Gender (M/F)	8/10	8/4	8/10
Age (years)	61.3 ± 2.21	53.92 ± 2.57	63.06 ± 1.39 ^1^
BMI (kg/m^2^)	27.19 ± 0.50	28.67 ± 1.01	29.23 ± 0.99
SBP (mmHg)	123.7 ± 4.18	146.08 ± 4.30 ^2^	131.3 ± 2.82 ^3^
DBP (mmHg)	74.4 ± 2.27	90.25 ± 3.65 ^4^	79.7 ± 2.01 ^5^

Values are the means ± SEM. ^1^ *p* = 0.006, vs. untreated hypertensive group. ^2^ *p* = 0.001, vs. normotensive group. ^3^ *p* = 0.014, vs. untreated hypertensive group. ^4^ *p* = 0.001, vs. normotensive group. ^5^ *p* = 0.017, vs. untreated hypertensive group.

## Supplementary Materials

The following are available online at https://www.mdpi.com/2077-0383/9/9/2764/s1, Figure S1: Circulating FGF21 levels in normotensive and hypertensive subjects, Figure S2: F2-isoprostane levels, Figure S3: Circulating lipid levels.
