# Supplementary material for: The Influence of Hypertensive Therapies on Circulating Factors: Clinical Implications for SCFAs, FGF21, TNFSF14 and TNF-α
Source: J Clin Med. 2020 Aug 26;9(9):2764. doi: 10.3390/jcm9092764 (PMC7576485; doi:10.3390/jcm9092764)
Supplement: Supplementary file 1 [file jcm-09-02764-s001.pdf]

Figure S1

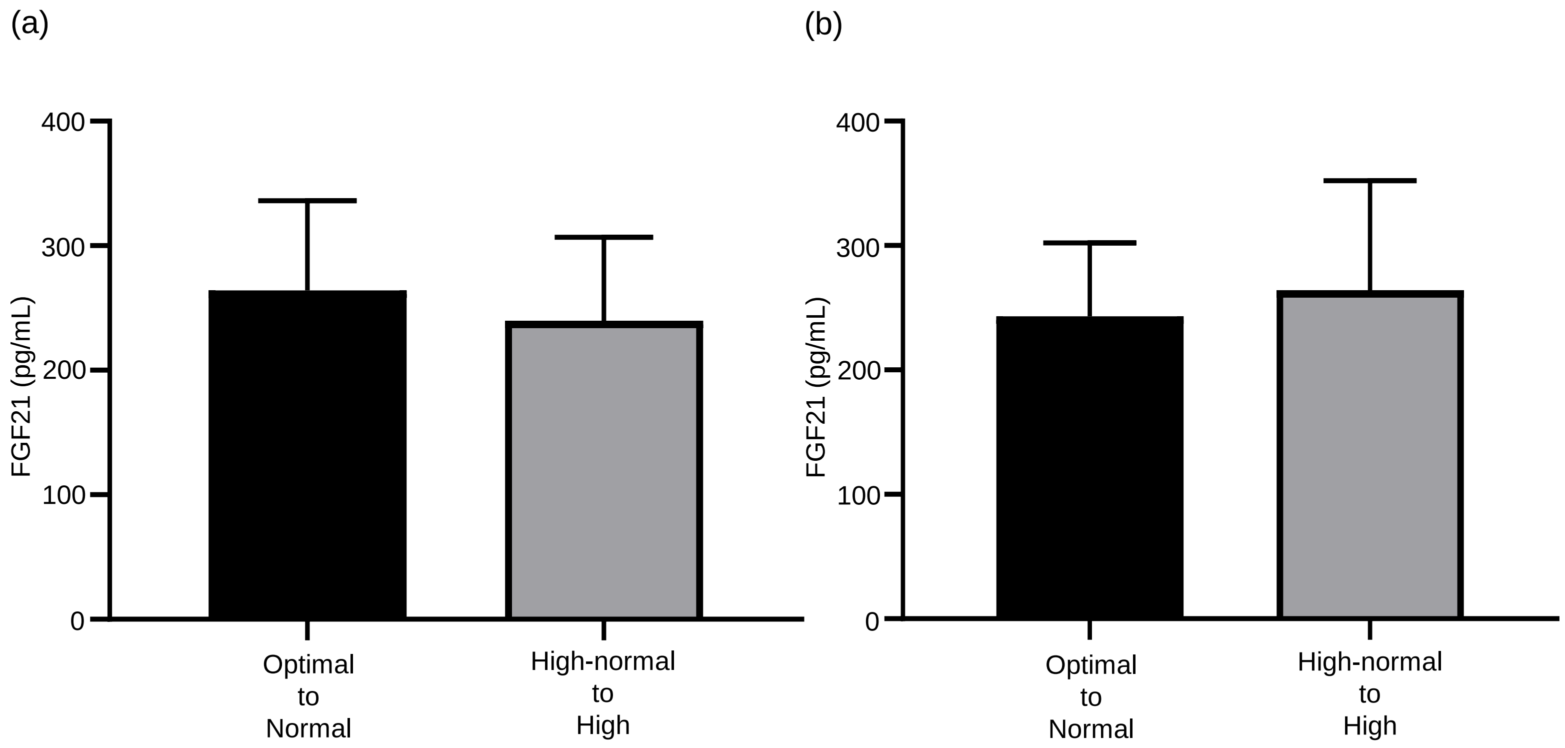

Figure S1. Circulating FGF21 levels in normotensive and hypertensive subjects (**a**) Circulating FGF21 levels in subjects with optimal to normal (≤129 mmHg) SBP or high-normal to high (≥130mmHg) SBP. (n=10-13) (**b**) Circulating FGF21 levels in subjects with optimal to normal (≤84 mmHg) DBP or high-normal to high (≥85mmHg) DBP. (n=8-15). All data presented as mean ± SEM.

Figure S2

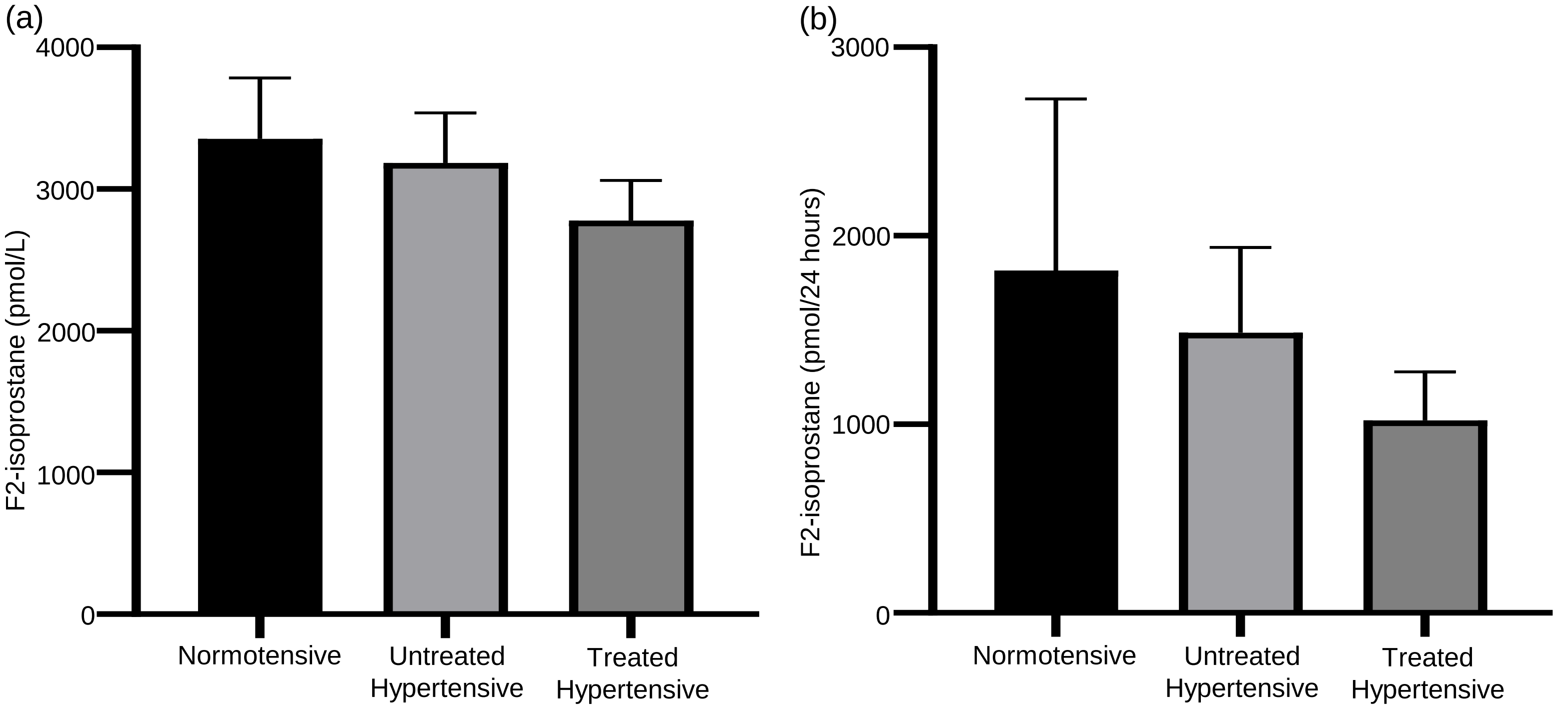

Figure S2. F2-isoprostane levels (**a**) Plasma F2-isoprostane concentration. n= 10-39 subjects/group. (**b**) Urine F2-isoprostane levels. n= 9-40 subjects/group. All data presented as mean ±SEM.

Figure S3

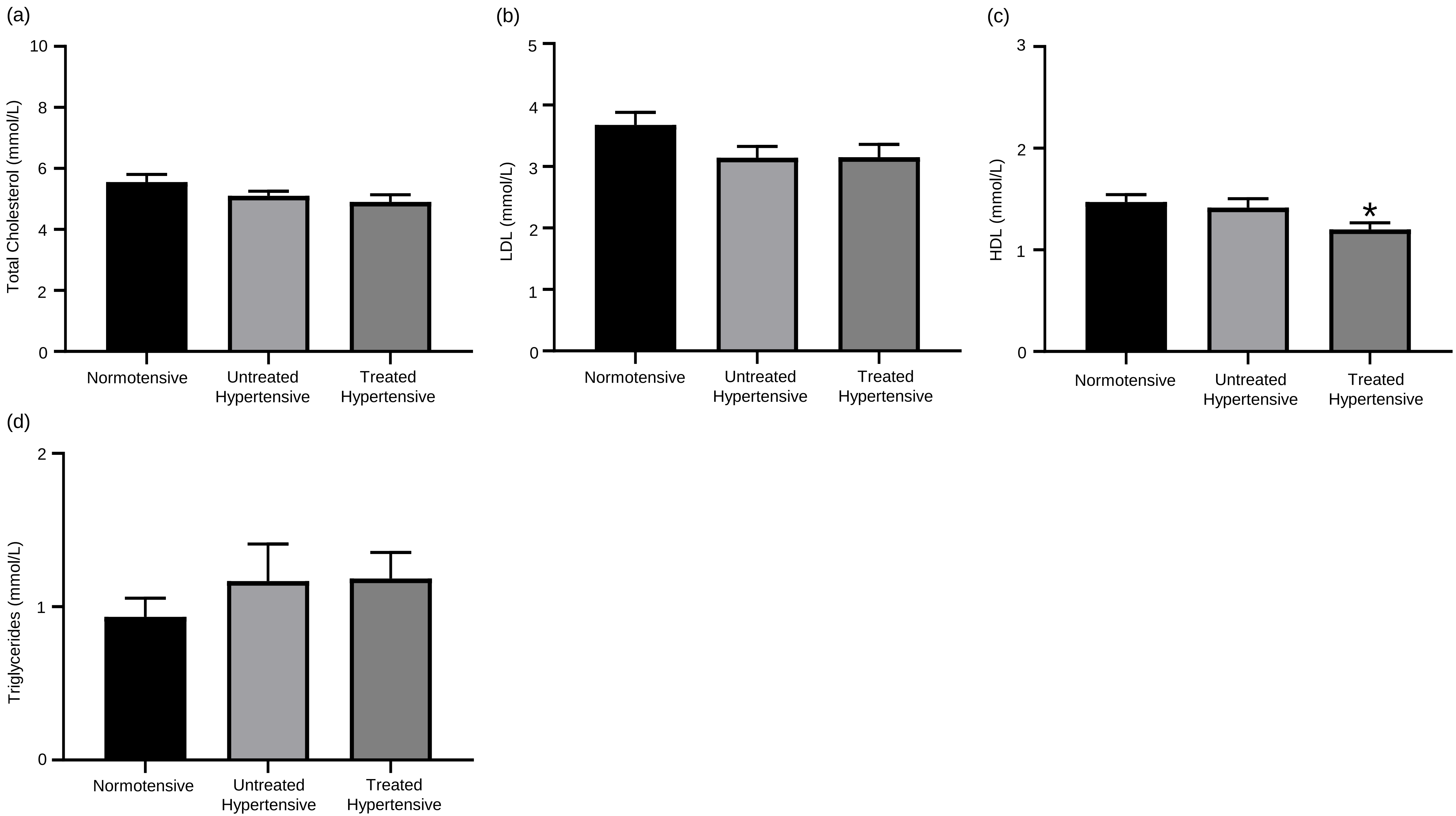

Figure S3. Circulating lipid levels (a) Total cholesterol levels. (b) LDL levels. (c) HDL levels. * Normotensive vs Treated Hypertensive p=0.025. (d) Triglycerides levels. n= 12-18 subjects/group, All data presented as mean ±SEM.
